# Non-acute chest pain in primary care; referral rates, communication and guideline adherence: a cohort study using routinely collected health data

**DOI:** 10.1186/s12875-022-01939-w

**Published:** 2022-12-22

**Authors:** Simone van den Bulk, Wouter A. Spoelman, Paul R. M. van Dijkman, Mattijs E. Numans, Tobias N. Bonten, Leiden University Medical Center(LUMC)

**Affiliations:** 1 Department of Public Health and Primary Care, Leiden University Medical Center, Postzone V0-P, Postbus 9600, 2300 RC Leiden, The Netherlands; 2 Department of Cardiology, Leiden University Medical Center, Postzone V0-P, Postbus 9600, 2300 RC Leiden, The Netherlands

**Keywords:** Chest pain, Primary care, Coronary artery disease, Communication, Referral rate, Guideline adherence, Routinely collected health data

## Abstract

**Background:**

The prevalence of coronary artery disease is increasing due to the aging population and increasing prevalence of cardiovascular risk factors. Non-acute chest pain often is the first symptom of stable coronary artery disease. To optimise care for patients with non-acute chest pain and make efficient use of available resources, we need to know more about the current incidence, referral rate and management of these patients.

**Methods:**

We used routinely collected health data from the STIZON data warehouse in the Netherlands between 2010 and 2016. Patients > 18 years, with no history of cardiovascular disease, seen by the general practitioner (GP) for non-acute chest pain with a suspected cardiac origin were included. Outcomes were (i) incidence of new non-acute chest pain in primary care, (ii) referral rates to the cardiologist, (iii) correspondence from the cardiologist to the GP, (iv) registration by GPs of received correspondence and; (v) pharmacological guideline adherence after newly diagnosed stable angina pectoris.

**Results:**

In total 9029 patients were included during the study period, resulting in an incidence of new non-acute chest pain of 1.01/1000 patient-years. 2166 (24%) patients were referred to the cardiologist. In 857/2114 (41%) referred patients, correspondence from the cardiologist was not available in the GP’s electronic medical record. In 753/1257 (60%) patients with available correspondence, the GP did not code the conclusion in the electronic medical record. Despite guideline recommendations, 37/255 (15%) patients with angina pectoris were not prescribed antiplatelet therapy nor anticoagulation, 69/255 (27%) no statin and 67/255 (26%) no beta-blocker.

**Conclusion:**

After referral, both communication from cardiologists and registration of the final diagnosis by GPs were suboptimal. Both cardiologists and GPs should make adequate communication and registration a priority, as it improves health outcomes. Secondary pharmacological prevention in patients with angina pectoris was below guideline standards. So, proactive attention needs to be given to optimise secondary prevention in this high-risk group in primary care.

**Supplementary Information:**

The online version contains supplementary material available at 10.1186/s12875-022-01939-w.

## Background

Chest pain often is the first symptom of coronary artery disease (CAD) and reason for the general practitioner (GP) to refer a patient to the cardiologist for additional diagnostic workup [1]. A distinction is made between acute chest pain and non-acute (stable) chest pain, because of their different pathophysiology, urgency and diagnostic pathway. Surprisingly, little is known about the incidence of non-acute chest pain in primary care, the referral rate and management of these patients. This is of high relevance because morbidity due to cardiovascular disease (CVD) is increasing and will increase further due to the ageing population and prevalence of cardiovascular risk factors [2, 3]. Therefore, it is important to optimise the use of available resources.

Stable angina pectoris (AP) is a clinical syndrome characterised by non-acute chest pain provoked by exertion or emotional stress and relieved by rest or nitrates [1]. It is caused by cardiac ischemia due to an insufficient supply of oxygen in patients with CAD [1, 4, 5]. CAD is a chronic, progressive disease and an independent risk factor for new acute cardiovascular events [1, 4, 5]. It requires long-term follow-up focussing on secondary prevention of acute cardiovascular events and symptom relief. The importance of cardiovascular risk management (CVRM) by treating existing modifiable risk factors is universally agreed upon and international guidelines are widely available [1, 5–8]. Despite this consensus, previous research suggests that physicians’ self-reported adherence in any domain to the guidelines is at best mediocre [9–12].

Many healthcare professionals are involved in the care of patients with chronic cardiac disease. The GP often functions as the coordinator of care in these patients [13]. Previous research showed that a well-established health care continuum reduces the risk of preventable adverse events and hospital (re)admissions [14, 15]. Proper communication between primary and secondary care is essential to establish a healthcare continuum [16–18]. Dutch Guidelines for communication between medical specialists and GPs indicate that correspondence should be sent to guarantee continuity of care as shortly as possible after discharge: within 5 days or anytime when a new diagnosis or treatment information becomes available [19]. Thereafter, the GP should summarise all incoming correspondence and link it to the patients relevant medical problem [20]. Previous research showed that only a quarter of Dutch GPs feel that communication from the specialist is received on time [21].

We aim to fill the research gap on the incidence, referral rate and management of patients with non-acute chest pain in primary care, by answering the following questions: What is the incidence of new, non-acute chest pain in primary care and how many of these patients are referred to secondary care? What proportion of the communication following referral, between the cardiologist and GP, could be considered adequate? What proportion of patients diagnosed with stable AP is treated according to pharmacological guideline recommendations?

## Methods

### Design and study population

We conducted an observational cohort study using anonymised routinely collected primary care health data. We extracted coded primary care Electronic Medical Record (EMR) data from GPs affiliated with – and sharing their registries in the STIZON data warehouse. STIZON acts as a trusted third party between the data sources and the research institute. STIZON is authorised by the data providers to manage and process the identifiable patient data. Before the database can be used for research the data is depleted of personal information that may be traced back to persons. The database covers a population of 1.49 million patients, aged 18 years and older, in the Netherlands and offers a representative sample of the Dutch population [22]. GPs register care data (e.g., patient contacts, diagnostic tests, medication, referrals) by ICPC (international classification of primary care), ATC (Anatomical Therapeutic Chemical code)– and referral codes [23, 24].

The study population consists of patients aged 18 years and older who contacted their GP for non-acute chest pain between January 2010 and January 2016, in whom the GP suspected a cardiac origin of the pain and used ICPC code K01 or K02 (heart pain and pressure/tightness of heart). We excluded patients with a cardiovascular history (ICPC codes K74 to K77) as the aim of our study is to evaluate the care pathway of new patients with non-acute chest pain. Only face-to-face contacts were included because administrative procedures, telephone consultation and laboratory results were considered irrelevant for our research question. In addition, patients with acute chest pain were excluded. These patients were identified by searching for terms that indicate acute chest pain in GP’s free text records at the index consultation (Acute coronary syndrome, unstable angina pectoris, ambulance, etc.; a complete list of exclusion terms is available as supplementary data (S1)).

### Data access and cleaning methods

The investigators had no access to the database used to create the dataset for analysis, containing personal data of the study population and free text. The primary selection was conducted by STIZON (trusted third party). STIZON selected contact moments registered as K01 or K02 between 2010 and 2016 and excluded patients with acute chest pain. A further selection of the study population was made by the investigators as described.

### Primary outcome

The primary outcome was the incidence of new, non-acute chest pain and was calculated per 1000 patient years of patients registered in the original database. For the denominator we used the number of patients registered in the original database times the length of the study period (6 years). The database is a dynamic cohort, with patients entering and leaving at a similar rate.

### Secondary outcomes

Referrals to the cardiologist were selected by outgoing correspondence to the cardiology department after the consultation for non-acute chest pain. The referral was considered a consequence of the consultation if it was within 1 month after the consultation. The proportion of referred patients was calculated by dividing the number of referred patients by the overall number of patients with non-acute chest pain.

To assess the communication between the cardiologist and GP, we examined incoming electronic correspondence from the cardiology department in the EMR. We selected patients referred between January 2010 and October 2015 to allow for 3 months of response time from the cardiology department. Only correspondence received within 3 months after referral was considered relevant. When multiple correspondence was available, only the first was analysed. We coded the conclusions of the cardiologist as registered by the GP into categories: CAD (ICPC codes K74 [Ischaemic heart disease with angina], K75 [Acute myocardial infarction] and K76 [Ischaemic heart disease without angina]), other cardiac disease (ICPC codes K70–73 and K77–84), and no cardiac disease (all other ICPC codes). When no ICPC-code was linked to the correspondence, we checked the ICPC-code of the index consultation after receiving the correspondence. If this was not coded K01 or K02, we assumed it was adapted due to the correspondence from the cardiologist and was therefore considered the conclusion of the cardiologist. Lastly, the start of a new relevant episode (ICPC-code K70-K84) within 1 month after receiving correspondence from the cardiology department was regarded as coding of the conclusion of the cardiologist.

To evaluate the quality of pharmacological CVRM in patients diagnosed with AP, we selected patients with a new ICPC code K74 (Ischaemic heart disease with angina) after the index consultation, between January 2010 and October 2015 to evaluate prescriptions up to 3 months after the diagnosis. We compared referred patients with non-referred patients. We assessed whether platelet aggregation inhibitors, statins, and antihypertensives were prescribed using their corresponding ATC codes (a complete list of used ATC codes is available as supplementary data (S2)). Medication prescriptions were considered relevant when prescribed within 3 months after the new diagnosis.

### Statistical analyses

Outcomes were analysed using descriptive statistics. Continuous variables are expressed as mean ± standard deviation (SD), and categorical data are presented as frequencies and percentages. Proportions were compared between referred and non-referred patients, using chi-square test and Fisher’s exact test as applicable. Results are reported as risk ratio’s. *P*-values < 0.05 were considered significant. All statistical analyses were performed using SPSS (version 23.0, IBM, Armonk, NY, USA).

## Results

### Incidence of non-acute chest pain

During the study period (2010–2016) a total of 10,341 patients contacted their GP for non-acute chest pain with a suspected cardiac origin. Of these patients, 9029 (87%) did not have a history of cardiovascular disease and comprise the final study population. Based on these numbers, the mean incidence of new, non-acute chest pain, suspected of a cardiac origin, was 1.01 per 1000 person years. The mean age of patients contacting their GP for non-acute chest pain was 60.4 years (SD 15.6), and 55% were women.

### Referral to the cardiologist and communication

**Fig. 1:**
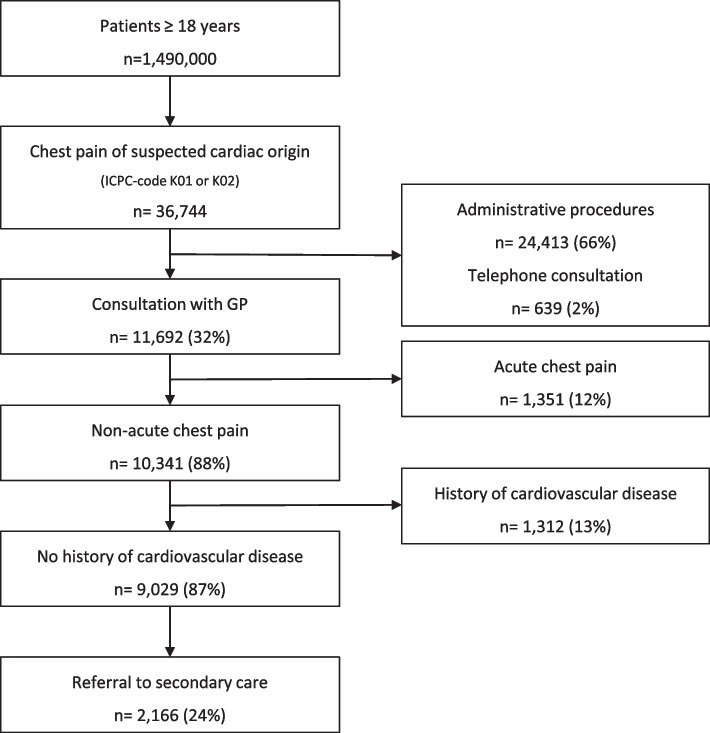
Selection of patients with new, non-acute chest pain in primary care between 2010 and 2016 suspected of cardiac disease

**Fig. 2:**
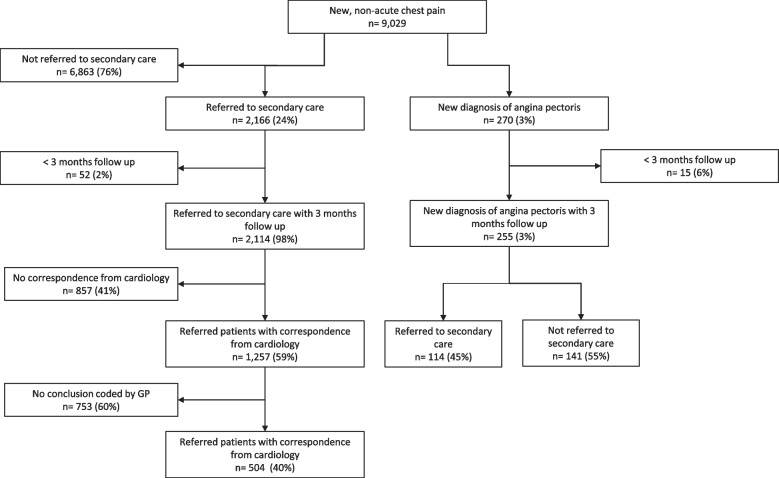
Selection of patients with angina pectoris and selection of correspondence. Allowing for 3 months of follow up

In total, 2166 (24%) patients were referred to the cardiologist (Fig. 1). For 857/2114 (41%) referred patients, the GP did not receive correspondence from the cardiologist within 3 months after referral. If correspondence was available, the GP did not code the conclusion from the cardiologist in the EMR in 753/1257 (60%) patients. For patients where correspondence was available, the GP coded CAD as the final diagnosis in 156/504 (31%) patients. Another cardiovascular disease was registered in 80/504 (16%) patients and no cardiovascular disease in 268/504 (53%) patients (Table 1) (Fig. 2).

**Table 1:** Received correspondence from cardiologists and coding of the conclusion in the electronic medical record (EMR) by general practitioners

	Number of patients (%)	
Referred to cardiologist^*^	2114	(100)
Correspondence from cardiologist not available	857	(41)
Correspondence from cardiologist available	1257	(59)
No conclusion in EMR	753	(60)
Conclusion registered in EMR	504	(40)
Coronary artery disease^¥^	156	(31)
Other cardiac disease	80	(16)
No cardiac disease	268	(53)
Muscular-skeletal disease	110	(22)
Respiratory disease	52	(10)
Gastro-intestinal disease	30	(6)
Psychosocial disease	6	(1)
Other	70	(14)

* Between January 2010 and November 2015, ¥ Coded as ICPC code K74 (Ischaemic heart disease with angina), K75 (Acute myocardial infarction) or K76 (Ischaemic heart disease w/o angina)

### CVRM

The GP diagnosed AP in 255/9029 (3%) patients after the consultation for new, non-acute chest pain (Fig. 2). 114 (45%) of these patients were referred to the cardiologist. Not all patients were treated according to pharmacotherapeutic guidelines. Of the 255 patients diagnosed with AP, 62 (24%) patients were not prescribed any antiplatelet therapy, of which 25 (37%) were prescribed oral anticoagulation (i.e., vitamin K antagonist or DOAC). 37/255 (15%) patients were not prescribed any form of antiplatelet therapy nor anticoagulation. No statin was prescribed in 69/255 (27%) patients. 67/255 (26%) patients were not treated with beta-blockers. Compared to patients who were referred to the cardiologist, patients not referred were less likely to be prescribed antiplatelet therapy: 67% vs 86% (RR 0.61 (95% CI 0.40–0.84)), a statin: 65% vs 83% (RR 0.62 (95% CI 0.42–0.85)) and beta-blockers: 69% vs 80% (RR 0.80 (95% CI 0.59–0.997)) (Table 2).

**Table 2:** Pharmacotherapeutic prescriptions by general practitioners for patients with angina pectoris within 3 months after diagnosis

			Number of patients (%)				
	Total		Referred		Not referred		*p*-value*
New diagnosis of angina pectoris	255	(100)	114	(45)	141	(55)	
Antiplatelet therapy	193	(76)	98	(86)	95	(67)	**0.001**
Acetylsalicylic acid/ Carbasalate calcium	187	(73)	95	(83)	92	(65)	**0.001**
Clopidogrel	57	(22)	35	(31)	22	(16)	**0.004**
Ticagrelor	18	(7)	7	(6)	11	(8)	0.634
Dipyridamol	3	(1)	1	(1)	2	(1)	1.000
Prasugrel	3	(1)	0	(0)	3	(2)	0.255
No antiplatelet therapy	62	(24)	16	(14)	46	(33)	
Vitamin K antagonist (VKA)	24	(39)	4	(25)	20	(43)	0.242
DOAC	1	(1)	0	(0)	1	(2)	1.000
No DOAC or VKA	37	(60)	12	(75)	25	(54)	0.237
Statin therapy	186	(73)	95	(83)	91	(65)	**0.001**
Antihypertensive therapy	240	(94)	109	(96)	131	(93)	0.361
Beta-blockers	188	(74)	91	(80)	97	(69)	**0.047**
Diuretics	67	(26)	25	(22)	42	(30)	0.156
Calcium channel blocker	66	(26)	32	(28)	34	(24)	0.473
Renin-angiotensin blocking agents	146	(57)	70	(61)	76	(54)	0.229
Nitrates	151	(59)	71	(62)	80	(57)	0.371

*p < 0.05 is considered significant

## Discussion

In our cohort study, the mean incidence of new, non-acute chest pain was 1.01/1000 patient-years. Three of every four patients (76%) were not referred to the cardiology department. The communication between cardiologist and GP was generally insufficient. The GP did not receive correspondence from the cardiologist within 3 months after referral in 41% of the referred patients. When correspondence was available, no conclusion was coded in the EMR by the GP in 60% of patients. Pharmacological treatment for patients diagnosed with AP was suboptimal; 15% were not prescribed antiplatelet nor oral anticoagulation therapy, 27% no statin, and 24% no beta-blocker.

### Incidence and referral rates

To our knowledge, there is no previous research available focussing on the incidence of new, non-acute chest pain in primary care. The majority of studies either focus on acute chest pain or do not discriminate between acute and non-acute chest pain, despite important differences in their care pathways [25–30]. The incidence in these previous studies were higher than in our study and ranged from 8.1 to 44.5 per 1000 person years. This could have several reasons. First, their inclusion criteria were broader, using additional (non-cardiac related) ICPC codes: ‘cardiovascular symptoms/complaints other’ (K29), ‘chest symptom/complaint’ (L04) and ‘pain respiratory system’ (R01) in addition to ‘heart pain’ (K01) and ‘pressure/tightness of heart’ (K02), thereby focusing more on the complaint chest pain in general and not necessarily suspected of cardiac origin. Second, they did not apply any additional exclusion criteria like we did. In our study, we excluded patients with a cardiovascular history, patients with acute chest pain, and contact moments for telephone calls and administrative procedures.

GPs referred 24% of the patients to the cardiologist for additional testing. The Dutch guideline for stable AP leading at the time advises to only refer patients to the cardiologist in case of an intermediate risk for significant CAD (atypical AP; 30–70%) in whom the GP is not able to do additional tests himself, patients with abnormal additional tests, known heart failure or insufficient effect of symptomatic treatment [31]. A previous Dutch study observed a referral rate of 35% in patients with chest pain, suspected of non-life-threatening cardiac disease, which is somewhat higher than our study finding [26]. This could be due to differences in the study populations: this previous study also included patients with a history of CVD, which could make GPs more inclined to refer, because patients with a history of CVD are at higher risk for new cardiovascular events.

### Communication and registration

After referring patients to secondary care, the GP received correspondence from the cardiology department within 3 months after referral for only 59% of these patients. In our opinion this is quite low, although a previous meta-analysis observed a similar percentage of 51–77% after 4 weeks, including studies between 1970 and 2005 [18]. Incoming email is automatically registered in the EMR, so an underestimation by non-registration at GP level seems unlikely. When correspondence was available, the GP did not adjust the conclusion in the EMR in 60% of patients. This percentage is even higher than the 30–40% missing diagnosis observed in a Dutch study assessing the quality of registration of cancer diagnosis [32, 33]. A study in the UK comparing primary care data to a hospital and disease registration observed a missing diagnosis for myocardial infarction in 30% of patients [34]. These percentages of unchanged or incorrect registration are lower than in our study.

GPs registered CAD as the final diagnosis in 31% of referred patients where correspondence from the cardiologist was available. This is in accordance with the 30% prevalence of non-life-threatening cardiac disease in patients with chest pain observed in a previous Dutch study. [26] These patients were referred to the cardiology department, but not the same day. Another Dutch study found AP to be the final diagnosis in only 18% of referred patients with non-acute chest pain [35]. Possibly, GPs are more inclined to adjust a conclusion when a diagnosis is found than when no aetiology is found, which results in an overestimation of the prevalence of CAD in our results.

### Guideline adherence

Guideline adherence in our cohort was suboptimal for antiplatelet therapy, statin therapy and beta-blockers. Similar results were found in previous research and were not limited to the Netherlands [36].

The Dutch guideline for CVRM for GPs (1st revision 2012), leading at the time, classifies all patients with known cardiovascular disease as high-risk patients [37]. According to these guidelines, treatment with antiplatelet therapy (acetylsalicylate) is indicated in patients with known CVD unless there is an indication for oral anticoagulation. Nonetheless, in our study, 37 (15%) patients diagnosed with AP did not receive any form of antiplatelet or anticoagulation therapy. This percentage is relatively low compared to a German cross-sectional study, where 47% of patients with CAD did not receive antiplatelet therapy [38]. At the same time it is considerably higher than the 7% observed in the EUROASPIRE registry, evaluating guideline adherence in patients with a history of either myocardial infarction or cardiovascular intervention (coronary artery bypass graft or percutaneous intervention), suggesting there is room for improvement [39]. Another Dutch study found a prescription rate for antiplatelet therapy of 78% in patients with type 2 diabetes and myocardial infarction [40]. Possibly, some patients had a strict contra-indication for antiplatelet therapy due to an increased risk of bleeding. However, the benefits of antiplatelet therapy substantially outweigh the risks of major bleeding complications in most patients [41, 42].

Every patient with a history of CVD should be treated with statin therapy irrespectively of the initial cholesterol or LDL level [37, 43]. In our cohort, only 73% of the AP patients were prescribed a statin, similar to the 70% found in a study in patients with type 2 diabetes and a history of myocardial infarction [40]. This is slightly lower than the 80% found in the EUROASPIRE registry, but higher than the 43% found in a German study [38, 39]. The Dutch guidelines on cardiovascular risk management were recently updated. Now even stricter LDL targets are used, stressing the importance of lipid-lowering treatment [44].

Lastly, the Dutch CVRM guideline, leading at the time, states that patients with known CVD should be treated with beta-blockers regardless of the systolic blood pressure. In the new Dutch guideline for AP in primary care, it is not considered standard therapy anymore, but together with calcium channel blockers it is still the first choice for symptomatic therapy [43]. This is in accordance with the European guideline for chronic coronary syndromes [1]. In our cohort, only 74% of patients were prescribed a beta-blocker. An explanation might be that GPs are either not familiar with the guideline’s content, consciously deviate from it, or that it is unclear who should take the initiative in prescription.

### Strengths and limitations

To our knowledge, this study is the first to give an insight into the incidence and care pathway for patients with new non-acute chest pain in primary care in the Netherlands. By using existing routine registries, we included a large number of patients and were not hampered by recall bias. Furthermore, we do not have missing data on the reason for contacting the GP, since registration of an ICPC in the EMR by the GP is obligatory in the Netherlands after every patient contact. Therefore we included every patient during the study period with chest pain as registered by the GP.

We are, however, well aware of some limitations of our research approach. First, using the existing registries, we analysed anonymised data that were not collected for research and therefore, do not provide additional patient characteristics and cardiovascular risk factors. Secondly, some selection bias is unavoidable, as the quality of the data depends on the registration quality of the GPs. Although registration of a diagnosis by GPs is obligatory, we cannot confirm the quality of this registration. We used strict selection criteria for chest pain in which GPs suspected a cardiac origin (K01 and K02). However, it is likely that GPs also use other general codes like ‘chest symptom/complaint’ (L04) or code directly as ‘ischaemic heart disease with angina ‘(K74). Therefore, there is a possibility that we underestimated the incidence of non-acute chest pain. In addition, we excluded telephone contacts and administrative procedures, so only patients seen by the GP were included. However, we have no reason to assume that the care pathway is different when the GP initially uses another code. Hence, we consider the results found on communication valid. This possible selection bias also applies to our selection of patients with a new AP diagnosis. The majority of these patients (55%) is not referred to secondary care. Therefore, it is uncertain whether the diagnosis CAD is confirmed or only suspected based on the clinical characteristics. This may have resulted in an underestimation of the pharmacological adherence in our cohort. Thirdly, we excluded patients with acute chest pain by searching for pre-defined free text terms as extensively as possible. However, it is possible that we did not exclude all acute chest pain patients and that patients with non-acute chest pain were accidentally excluded. This may result in either an under-or overestimation of the incidence and referral rates, depending on the ratio of misclassification of non-acute versus acute patients.

Lastly, it is important to note that routinely collected health data are increasingly used for epidemiological purposes. However, the suboptimal registration of diagnosis can lead to significant under- or overestimation of disease prevalence. For example, a study assessing the quality of cancer registration in Dutch primary care, showed that 30–40% of cancer diagnosis can be missed when using coded routinely collected primary health care data, while at the same time up to 130% can be false-positive [32, 33]. Special attention needs to be paid before using these data and it might be necessary to validate the data with external registries.

### Implications

By creating insight into the care pathway of patients with new, non-acute chest pain, we identified opportunities for further research to improve the quality of care for these patients. We believe these results are also relevant for countries with similar healthcare systems as the Netherlands, where the GP functions as a gatekeeper between primary and secondary care and (non-) invasive testing for CAD is only possible through referral to the cardiologist.

The outcomes of this study confirm that guideline adherence for cardiovascular risk management is substandard and there is room for improvement. Both cardiologists and GPs need to improve the communication and registration in referred patients to ensure a continuum of care and eventually reduce hospital admissions and adverse events [14, 15]. After referral, GPs should actively confirm that patients with CAD receive optimal secondary prevention to minimise the risk for a new cardiovascular event. Population health management software can possibly help the GP to identify these patients [45]. Additionally, comprehensive community based interventions could offer new, promising strategies to reduce cardiovascular risk factors by tackling multiple health care barriers and involving patients, physicians and non-physician health workers [46, 47].

## Conclusion

New, non-acute chest pain is a commonly presented reason for encounter in general practice. The majority of patients is not referred to secondary care. Communication and registration of the final diagnosis are suboptimal for referred patients and require attention and improvement from both cardiologists and GPs. We found secondary pharmacological prevention in patients diagnosed with stable AP to be below standard in current care, at least on the level of registration. These results highlight the ongoing need to optimise the care pathway in patients with non-acute chest pain.

## Supplementary Information

**Additional file 1.**

**Additional file 2.**

## Data Availability

The data that support the findings of this study are available from STIZON but restrictions apply to the availability of these data, which were used under license for the current study, and so are not publicly available. Data are however available from the authors upon reasonable request and with permission of STIZON.

## Abbreviations

AP: Angina Pectoris
ATC: Anatomical Therapeutic Chemical code
CAD: Coronary Artery Disease
CVD: Cardiovascular Disease
CVRM: Cardiovascular Risk Management
EMR: Electronic Medical Record
GP: General practitioner
ICPC: International Classification of Primary Care
