# Supplementary material for: Non-acute chest pain in primary care; referral rates, communication and guideline adherence: a cohort study using routinely collected health data
Source: BMC Prim Care. 2022 Dec 22;23:336. doi: 10.1186/s12875-022-01939-w (PMC9784001; doi:10.1186/s12875-022-01939-w)
Supplement: Supplementary file 1 — Additional file 1. [file 12875_2022_1939_MOESM1_ESM.pdf]

Supplemental data 1

Exclusion terms in electronic medical record

hartinfarct;
hartinfarkt;
infarct;
infarkt;
myocardinfarct;
myocardinfarkt;
iap;
i.a.p.;
instabiel;
instabiele;
acs;
cave acs;
acuut coronair;
coronair syndroom;
acute coronary;
coronary syndrome;
acute coronair;
coronaire syndroom;
nstemi;
stemi;
ehh;
eerst hh;
eerste hh;
harthulp;
hart hulp;
eerste hart hulp;
eerste harthulp;
eersteharthulp;
1e harthulp;
1e hart hulp;
seh;
ehbo;
spoedeisend;
spoedeisende hulp;
centrum eerste hulp;
ceh;
ambu;
ambulance;
ambulancerit;
ccu.

With exception of:

angst infarct;
angst voor infarct;
bang voor infarct;
angst hartinfarct;
angst voor hartinfarct;
bang voor hartinfarct;
geen infarct;
geen aanwijzing infarct;
infarct onwaarschijnlijk;
infarct zeer onwaarschijnlijk;
geen hartinfarct;
geen myocardinfarct;
geen aanwijzing hartinfarct;
geen aanwijzing myocardinfarct;
hartinfarct onwaarschijnlijk;
hartinfarct zeer onwaarschijnlijk;
herseninfarct;
hersen infarct;
niet instabiel;
geen iap;
geen aanwijzing iap;
niet instabiel;
geen instabiele;
acs uitgesloten;
acs onwaarschijnlijk;
acs zeer onwaarschijnlijk;
geen acs;
geen verdenking acs;
geen aanwijzing acs;
geen aanwijzingen acs;
geen aanwijzingen voor acs;
geen aanwijzing voor acs;
geen acuut coronair;
geen coronair syndroom;
geen aanwijzingen voor acuut coronair;
geen aanwijzingen acuut coronair;
geen aanwijzing voor acuut coronair;
geen aanwijzing acuut coronair;
geen stemi;
geen aanwijzing stemi;
geen aanwijzing voor stemi;
geen nstemi;
geen aanwijzing nstemi;
geen aanwijzing voor nstemi;
geen indicatie voor spoed;
geen indicatie spoed;
geen indicatie ehh;
geen indicatie voor eerste hart hulp;
geen indicatie voor ehh;
geen indicatie 1e hart hulp;
geen indicatie 1e harthulp;
geen indicatie voor 1e harthulp;
geen indicatie voor 1e hart hulp;
niet naar ehh;
niet naar seh;
niet naar eerste hart hulp;
niet naar eerste harthulp;
niet naar 1e hart hulp;
niet naar 1e harthulp;
geen ambu;
geen ambulance;
niet met ambulance;
niet met ambu;
ambulant;
accu.
