# Supplementary material for: Non-acute chest pain in primary care; referral rates, communication and guideline adherence: a cohort study using routinely collected health data
Source: BMC Prim Care. 2022 Dec 22;23:336. doi: 10.1186/s12875-022-01939-w (PMC9784001; doi:10.1186/s12875-022-01939-w)
Supplement: Supplementary file 2 — Additional file 2. [file 12875_2022_1939_MOESM2_ESM.pdf]

Supplemental data 2 – ATC codes used for medication prescriptions

Anticoagulants
B01AA04
B01AA07
B01AC04
B01AC06
B01AC07
B01AC08
B01AC22
B01AC24
B01AC56
B01AF01
B01AF02
B01AF03
B01AE07
C10BX01
C10BX02
C10BX04
C10BX06
C10BX08

Statins
C10*

Antihypertensive agents
C03*
C07*
C08*
C09*
C01DA*
